# Neural Correlates of Amusia in Williams Syndrome

**DOI:** 10.3390/brainsci4040594

**Published:** 2014-11-21

**Authors:** Miriam D. Lense, Nathan Dankner, Jennifer R. Pryweller, Tricia A. Thornton-Wells, Elisabeth M. Dykens

**Affiliations:** 1 Vanderbilt Kennedy Center, Vanderbilt University, Nashville, TN 37203, USA; E-Mails: nathan.dankner@vanderbilt.edu (N.D.); t.thornton-wells@vanderbilt.edu (T.A.T.-W.); elisabeth.dykens@vanderbilt.edu (E.M.D.); 2 Psychology and Human Development, Vanderbilt University, Nashville, TN 37203, USA; 3 Emory University School of Medicine, Marcus Autism Center, Children’s Healthcare of Atlanta, Atlanta, GA 30329, USA; 4 Department of Radiological Sciences, St. Jude Children’s Research Hospital, Memphis, TN 38105, USA; E-Mail: jennifer.pryweller@stjude.org; 5 Vanderbilt University Institute of Imaging Science, Vanderbilt University, Nashville, TN 37203, USA; 6 Center for Human Genetics Research, Vanderbilt University, Nashville, TN 37203, USA

**Keywords:** Williams syndrome, amusia, music, DTI, MRI, superior longitudinal fasciculus, pars orbitalis

## Abstract

Congenital amusia is defined by marked deficits in pitch perception and production. Though historically examined only in otherwise typically developing (TD) populations, amusia has recently been documented in Williams syndrome (WS), a genetic, neurodevelopmental disorder with a unique auditory phenotype including auditory sensitivities and increased emotional responsiveness to music but variable musical skill. The current study used structural T1-weighted magnetic resonance imaging and diffusion tensor imaging to examine neural correlates of amusia in 17 individuals with WS (4 of whom met criteria for amusia). Consistent with findings from TD amusics, amusia in WS was associated with decreased fractional anisotropy (FA) in the right superior longitudinal fasciculus (SLF). The relationship between amusia and FA in the inferior component of the SLF was particularly robust, withstanding corrections for cognitive functioning, auditory sensitivities, or musical training. Though the number of individuals with amusia in the study is small, results add to evidence for the role of fronto-temporal disconnectivity in congenital amusia and suggest that novel populations with developmental differences can provide a window into understanding gene-brain-behavior relationships that underlie musical behaviors.

## 1. Introduction

As a complex auditory stimulus, music engages multiple brain areas and networks and can be a window into understanding neural connectivity (e.g., [1]). Recent years have seen a growing interest in the neural underpinnings of musical behaviors, including the effects of musical training on neurodevelopment [2] and the neuroanatomy of individuals with either enhanced or impaired musical abilities. For example, congenital amusia, which affects ~2.5% of otherwise typically developing (TD) individuals, involves marked deficits in pitch perception and production despite normal cognitive abilities and musical exposure [3,4]. Amusics are unable to perceive pitch deviations below one semitone [5] or recognize out-of-tune notes in otherwise familiar melodies [6]. Pitch impairments in congenital amusia are likely due to decreased functional and structural connectivity between the right temporal and inferior frontal cortices [7,8,9,10].

Congenital amusia and its neural correlates have historically only been studied in TD adult populations without cognitive impairments. While this work has been an important first step in understanding the neural architecture underlying amusia, expanding the lens to other populations such as children and those with developmental differences can enhance our knowledge about how and why amusia develops. In contrast to acquired amusia (for example [11,12]), which results from a brain lesion or insult that impairs previously developed musical skills, in congenital amusia, pitch perception fails to appropriately develop due to atypical brain development [13]. Twin and family studies point to a genetic component to congenital amusia, as well [6,14]. Under this framework, amusia has recently been reported in a group of otherwise cognitively intact children [13]. Similar to studies in amusic adults [15,16], in children with amusia, event-related potentials (ERPs) to a tone series indicated intact initial auditory processing via the N100, mismatch negativity (MMN) and P200 components, but impaired conscious detection of deviant tones via the P300 [13]. Hence, even at this earlier developmental time point, support was found for the idea that amusia is due to disconnectivity along the fronto-temporal cortical pathway rather than isolated impairments in primary auditory cortex [7,8,9,10,13].

Further knowledge about amusia can also come from patient populations with atypical developmental histories. Amusia has recently been identified in Williams syndrome (WS; [17]), a neurodevelopmental disorder caused by the deletion of ~28 genes on chromosome 7 [18]. WS is associated with a unique cognitive-behavioral phenotype including mild to moderate cognitive impairment, a weakness in spatial abilities, generalized anxiety and phobias, attention problems, and hypersociability (see [19] for a review). Of relevance to the field of music perception and cognition, people with WS also have a unique auditory phenotype, which includes higher rates of auditory aversions, fascinations, hyperacusis, and odynacusis compared to TD individuals and those with other developmental disabilities [20]. In addition, compared to TD controls or peers with other developmental disabilities, those with WS report a greater emotional responsiveness to music [21,22]. Despite their auditory sensitivities and a strong interest in music, musical skill is actually quite variable in WS, and rates of amusia were found to be higher in WS than in the TD population [17]. Furthermore, in this large previous study of children and adults with WS, amusia was primarily predicted by exposure to musical training, rather than cognitive abilities, sound sensitivities, musical engagement (*i.e.*, time spent listening to or playing music), or low-level auditory processing [17].

However, it remains unknown if the neural correlates of amusia in individuals with WS are the same as in TD individuals with amusia. If they are consistent, this provides further support for the appropriateness and utility of examining amusia in populations with developmental disabilities (who are traditionally excluded from studies of amusia). Furthermore, consistency across populations would be evidence for the robustness of the neural network associated with pitch processing impairments. In addition to being a well-characterized developmental disorder on a behavioral level, the neuroanatomy of WS suggests that the neural correlates of amusia in WS may mirror those seen in TD individuals with amusia.

There have been very few prior studies examining neural correlates of musicality in WS and these studies have typically focused only on the auditory cortex. Histological and neuroimaging studies have reported relatively preserved or enlarged auditory cortex volumes in WS *versus* TD participants (with heterogeneity among findings likely due to differences in sample characteristics or imaging or segmentation processing parameters (e.g., [23,24,25,26,27]). Researchers have hypothesized that this relative preservation of auditory cortex volume might underlie the relative auditory strengths in WS. However, amusia in TD is not typically associated with volumetric differences in the auditory cortex, as well [7] (though see [10] for evidence otherwise).

In contrast, amusia in TD individuals is linked to decreased white matter volume in fronto-temporal tracts such as the arcuate fasciculus, which connects the right inferior frontal gyrus (IFG) and right auditory cortex [8]. Reduced white matter has also been reported in the pars orbitalis region of the right IFG in amusia, with white matter concentration correlating with performance on pitch-based tasks [7]. Compared to the TD brain, the WS brain shows reduced white matter volume in the frontal lobe [23,28]. Neuroimaging studies of diffusion in the WS brain have reported atypical directionality of fibers in the right superior longitudinal fasciculus (SLF; [29]). The SLF is a major white matter association pathway that connects the parieto-temporal association areas and the frontal lobe and includes the arcuate fasciculus [30]. Fractional anisotropy (FA), which quantifies directional diffusion at each voxel [31] and relates to structural connectivity and the efficiency of neuronal signal transmission [32], has been reported to be increased in the right SLF in WS compared to TD individuals and to those with developmental delay [33,34].

In the current paper, we present the first neuroanatomical evidence for amusia in WS. Based on the findings of amusia in TD individuals, we used a top-down, *a priori* region-of-interest-based approach, restricting our analyses to the brain regions that have previously been examined as possible substrates of amusia in the TD population (*i.e.*, primary auditory cortex (transverse temporal gyrus, superior temporal gyrus), pars orbitalis of the IFG, and SLF [7,8,9,10,35,36]). We hypothesized that amusia in WS would be associated with reduced white matter volume in the right IFG but not in the auditory cortex. We further hypothesized that amusia in WS would be associated with decreased structural connectivity along the right fronto-temporal pathway, as measured by FA in the SLF. As studies of gray matter differences between TD amusics and non-amusics have yielded conflicting results [7,10,36], we did not have *a priori* hypotheses regarding the relationship between amusia in WS and gray matter volume in the IFG and auditory cortex. Specifically, while some studies have reported increased gray matter volume in the right (but not left) IFG [7,10], others have reported decreased gray matter volume in left (but not right) IFG [36]. In regards to auditory cortex, studies have reported decreased right side gray matter in amusics [10], decreased left side gray matter in amusics [36], and no gray matter volume differences between amusics and controls [7]. We conducted our analyses in both the right and left hemispheres. However, since the majority of studies in TD amusia implicate the right and not the left hemisphere, we hypothesized that the neural correlates of amusia in WS would only be evident in the right hemisphere [7,8,35]. Indeed, despite the different results reported in regards to gray matter volume, studies using other neuroimaging techniques such as functional imaging [9] or cortical thickness [35], have implicated impairments primarily in the right fronto-temporal network.

## 2. Experimental Section

### 2.1. Participants

Participants included 17 individuals with genetically-confirmed WS who attended a residential, university-based music research camp. Although camp activities focused on music, musical experiences or skills were not required to attend the camp. Participants represented a subset of individuals from a larger behavioral study on amusia in WS [17] who also consented to participate in a neuroimaging study. All participants were reported to have normal hearing. The study was approved by the Vanderbilt University Institutional Review Board (IRB; project codes 040544 and 120543) and conducted following the rules of the Declaration of Helsinki of 1975. Parents/guardians of participants provided informed written consent while participants provided written assent in accordance with the procedures of the Vanderbilt University IRB.

Participants completed the Kaufman Brief Intelligence Test, 2nd Edition (KBIT-2; [37]) as a screener of their cognitive functioning. Parents of participants completed the Edinburgh Handedness Inventory [38] and the Musical Background Questionnaire [17,39]. Two measures of musical training were examined in the current study: (1) duration of cumulative years of formal individual extracurricular training; and (2) exposure to different types of musical training (*i.e.*, experience with individual or group lessons through school or extracurricular activities, as well as ensemble participation, for various instruments). While research in TD individuals has typically used the former (*i.e.*, duration of individual music lessons) as an index of musical training (e.g., [40]), the latter metric (*i.e.*, exposure to different lesson types) appears to be a better predictor of musical abilities in WS [17]. This is likely because individuals with developmental disabilities do not typically have the same opportunities for standard individualized musical training experiences. Parents also completed the Sensitivity to Sounds questionnaire [17,39], which assesses how bothered or frightened their child is to 21 specific non-musical sounds (e.g., clock ticking, thunder, *etc.*) and five different sound characteristics (loudness, suddenness, duration, low pitched, high pitched) on a seven-point Likert scale. Total scores were calculated to characterize participants’ sensitivity to specific sounds and sound characteristics, respectively, in order to assess general (non-musical) auditory sensitivities. (The Sensitivity to Sounds questionnaire has been collected in 49 TD participants in our laboratory. TD participants report significantly lower sensitivity to specific sounds (37.7 ± 18.6) and sound characteristics (13.8 ± 6.3) than participants with WS (*p*’s < 0.05), consistent with previous studies examining sound sensitivities in WS *versus* TD populations (e.g., [20,41]).) Demographic, IQ and musical background information can be found in Table 1.

**Table 1:** Demographic and musical background information (Mean ± SD (range)).

Variable	Total Sample (*n* = 17)	Non-amusics (*n* = 13)	Amusics (*n* = 4)
Age (years)	26.1 ± 9.1 (16–48)	25.1 ± 10.0 (16–48)	29.3 ± 4.9 (23–34)
Gender	12 males, 5 females	10 males, 3 females	2 males, 2 females
Full Scale IQ	70.3 ± 17.4 (43–93)	74.4 ± 15.2 (46–93)	57.0 ± 19.8 (43–86)
Handedness	0.6 ± 0.7 (−1–+1)	0.52 ± 0.75 (−1–+1)	0.87 ± 0.26 (0.48–1)
DTT score	20.9 ± 4.3 (12–26)	22.8 ± 2.3 (20–26)	14.5 ± 2.4 (12–17)
Cumulative years of private extra-curricular training	9.2 ± 11.9 (0–46)	10.2 ± 12.5 (0–46)	5.9 ± 10.5 (0–21.5)
Number of lesson types	3.4 ± 1.8 (0–6)	3.9 ± 1.7 (0–6)	1.8 ± 1.0 (1–3)
Time play music currently (hours)	1.4 ± 1.7 (0–6)	1.4 ± 1.6 (0–6)	1.7 ± 2.2 (0–5)
Time listen music currently (hours)	2.7 ± 2.1 (0.5–8)	2.7 ± 2.2 (0.5–8)	2.9 ± 2.3 (1–6)
Age began private music lessons (years)	11.7 ± 3.2 (6–19 years; *n* = 14)	11.6 ± 3.3 (6–19 years; *n* = 12)	12.5 ± 3.5 (10–15 years; *n* = 2)
Sensitivity to specific (non-musical) sounds	49.6 ± 21.1 (24–102)	47.1 ± 21.0 (24–102)	57.8 ± 22.2 (26–78)
Sensitivity to sound characteristics	18.1 ± 6.3 (5–29)	18.0 ± 7.0 (5–29)	18.3 ± 4.6 (14–24)

DTT: Distorted Tunes Test.

Participants individually completed the Distorted Tunes Test (DTT; [6]) as a measure of pitch amusia. The DTT has been documented to be a reliable measure of amusia in the WS population [17]. (While studies in TD individuals commonly use the Montreal Battery of Evaluation of Amusia (MBEA [42,43]), the DTT is a better fit for WS because it uses highly engaging stimuli and does not have the same working memory requirements of the MBEA, which is a same/difference task of novel melodies [17]. Auditory working memory is a relative weakness in WS [44,45] such that the MBEA would have introduced an additional cognitive confound. (An online version of the MBEA has been proposed that does not have this working memory requirement [46].) Work in our laboratory indicates that performance on the DTT in WS is not related to auditory short term or auditory working memory as measured by the Wechsler Adult Intelligence Scale, 4th edition (WISC-IV; [47]) Digit Span task (*r*’s ≤ 0.2, *p*’s > 0.2) [48]. The DTT’s method of testing pitch detection has been termed “diagnostic” of pitch amusia ([43], p. 250).) In brief, participants heard 26 well known tunes (e.g., children’s folk songs, holiday tunes, patriotic fare), 17 of which contained note errors [6]. Participants indicated if each tune was played correctly or incorrectly via verbal response or by pointing to a happy or sad face. The tunes were presented at 68 dB from two speakers approximately 40 cm in front of the participant. In the current sample, scores were broadly distributed and ranged from chance level to perfect score (12–26). The mean DTT score was 20.9 (80.4% correct) with a standard deviation of 4.3. Four individuals (23.5%) were considered amusic due to meeting criteria for amusia on the DTT (scores ≤ 18). (A convenience sample of 36 TD participants has completed the DTT under our testing conditions, with one individual (2.8%) meeting criteria for amusia, consistent with the previous literature [3,4].)

### 2.2. Neuroimaging Data Acquisition

A high-resolution T1-weighted anatomical volume (TR = 4.6 ms, TE = 9 ms, FOV = 256 mm^2^, 1 mm isotropic voxels, 170 sagittal slices, 6 min 30 s duration) was acquired and used as a template for image registration. Diffusion weighted data were collected using a high-angular resolution diffusion imaging (HARDI) sequence (2.5 mm^2^ isotropic voxels; 50 axial slices; 92 diffusion directions, *b* = 1600 s/mm^2^, T2-weighted volume, *b* = 0 s/mm^2^; 14 min 34 s). HARDI has the ability to resolve multiple fibers crossing through a single voxel, increasing the sensitivity of diffusion-based diffusion tensor imaging (DTI) measures [49,50]. Neuroimaging was performed on a 3 Tesla Philips Achieva MR scanner located at the Vanderbilt University Institute of Imaging Science.

### 2.3. Surface Reconstruction and Volumetric Calculation

**Figure 1:**
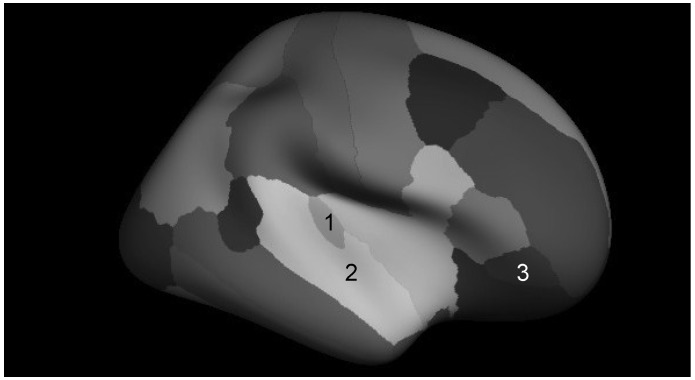
Cortical parcellation of the target regions of interest. (**1**) Transverse temporal gyrus; (**2**) Superior temporal gyrus; (**3**) Pars orbitalis.

The Freesurfer image analysis suite (http://surfer.nmr.mgh.harvard.edu/) was used for cortical reconstruction and volumetric segmentation. Processing of the T1-weighted images in Freesurfer included motion correction [51], removal of non-brain tissue using a hybrid watershed/surface demonstration procedure [52], automated Talairach transformation, white matter segmentation of subcortical volumetric structures [53,54], intensity normalization [55], tessellation of the gray matter/white matter boundary, automated topology correction [56,57], and surface deformation following intensity gradients to optimally place the gray/white and gray/cerebrospinal fluid borders at the location where the greatest shift in intensity defines the transition to the other tissue class [58,59,60]. The cortex was parcellated into units based on gyral and sulcal structure [61,62], and estimates of gray and white matter volume were derived for the pre-specified regions of interest (ROIs) of the right and left transverse temporal gyri, superior temporal gyri, and pars orbitalis (see Figure 1). Freesurfer morphometric procedures have shown good test-retest reliability across scanner manufacturers and across field strengths [63,64].

### 2.4. DTI Image Processing and Analysis

**Figure 2:**
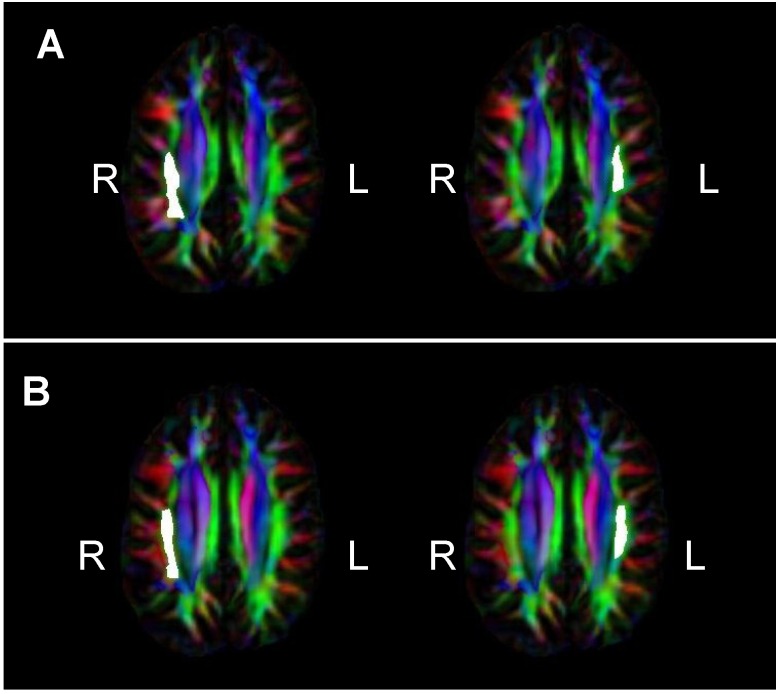
Superior longitudinal fasciculus (SLF) regions of interest (ROIs) selected in Reproducible Objective Quantification Scheme (ROQS), shown on fractional anisotropy (FA) map of representative Williams syndrome participant. White matter fiber tract directionality is given by the FA map: red (right-left), green (anterior-posterior), blue (superior-inferior). (**A**) Right and left superior SLF ROIs; (**B**) Right and left inferior SLF ROIs.

Images were visually inspected for artifacts and underwent quality assurance and preprocessing procedures. Using the FMRIB Software Library (FSL; [65,66]), raw HARDI images were eddy current and motion corrected. Then, each subject’s HARDI and T1W images were skull stripped. Rigid registration was used to co-register the HARDI image to the ACPC oriented T1W image collected during the same scan session. In DTI Studio, tensor fit and pixel-based outlier rejection were performed using the following threshold criteria: “Minimum bad area” = 80 (30 pixels per 1 mm^2^), “Minimum *Z*-value” = 2 (standard deviations from global mean signal), “Minimum B0-Value” = 100 (intensity threshold to remove floor noise). For each subject, the tensor fit produced output files that were used to calculate fractional anisotropy in *a priori* white matter fiber tract regions of interest (ROIs) in Reproducible Objective Quantification Scheme (ROQS), a software-based tool for determining regional white matter measurements of diffusion tensor imaging parameters [67]. The technique exploits fiber information from the diffusion tensor to segment anatomically distinct white matter fiber tracts for quantitative diffusion tensor imaging analysis. ROIs were delineated on a best-fit 2D slice, per the ROQS manual. Bilateral ROIs were segmented individually for each hemisphere. We obtained measures of fractional anisotropy from four ROIs: the inferior and superior portions of the superior longitudinal fasciculus (SLF), bilaterally (see Figure 2). ROQS has previously been used to assess white matter integrity in WS and demonstrated results consistent with those from tract-based spatial statistics [33].

### 2.5. Statistical Analyses

Raw data can be found in the Supplementary Table S1. Preliminary analyses revealed that DTT performance was not related to age (*r* = −0.008, *p* = 0.978), gender (*r* = −0.322, *p* = 0.207), handedness (*r* = −0.109, *p* = 0.678), IQ (*r* = 0.370, *p* = 0.144), sensitivity to specific (non-musical) sounds (*r* = −0.215, *p* = 0.406), or sensitivity to sound characteristics (*r* = −0.071, *p* = 0.787). DTT was not significantly related to cumulative years of individual musical training (*r* = 0.321, *p* = 0.121) but was strongly related to the number of types of music lesson experiences (*r* = 0.721, *p* = 0.001).

To examine brain areas related to DTT, zero-order correlations were conducted between DTT scores and the gray and white matter volumes of the right and left transverse temporal gyri, superior temporal gyri, and pars orbitalis. To examine structural connectivity, correlations were conducted between DTT scores and FA of right and left inferior and superior segments of the SLF. Based on results in TD individuals, we hypothesized that lower DTT scores (consistent with amusia) would be associated with reduced white matter volume in the right pars orbitalis and reduced FA in the right inferior and superior SLF. We then conducted partial correlations to examine if correlations remained significant when controlling for potential confounding factors of age, IQ, handedness, sound sensitivities, and musical training. Results did not change when controlling for age or handedness.

As many studies of amusia in TD individuals have utilized a between-group rather than continuous design, we similarly conducted dichotomous analyses comparing volume and FA of these same brain areas in individuals with WS with, *versus* without, amusia via Mann-Whitney U tests. Given the small sample size, dichotomous analyses should be considered exploratory. The four individuals with amusia had fewer types of music lessons than those without amusia but did not differ in respect to age, handedness, IQ, gender, sound sensitivities, or other musical engagement variables (*i.e.*, time spend listening or playing music, age started private music lessons, cumulative years of music lessons).

## 3. Results

**Figure 3:**
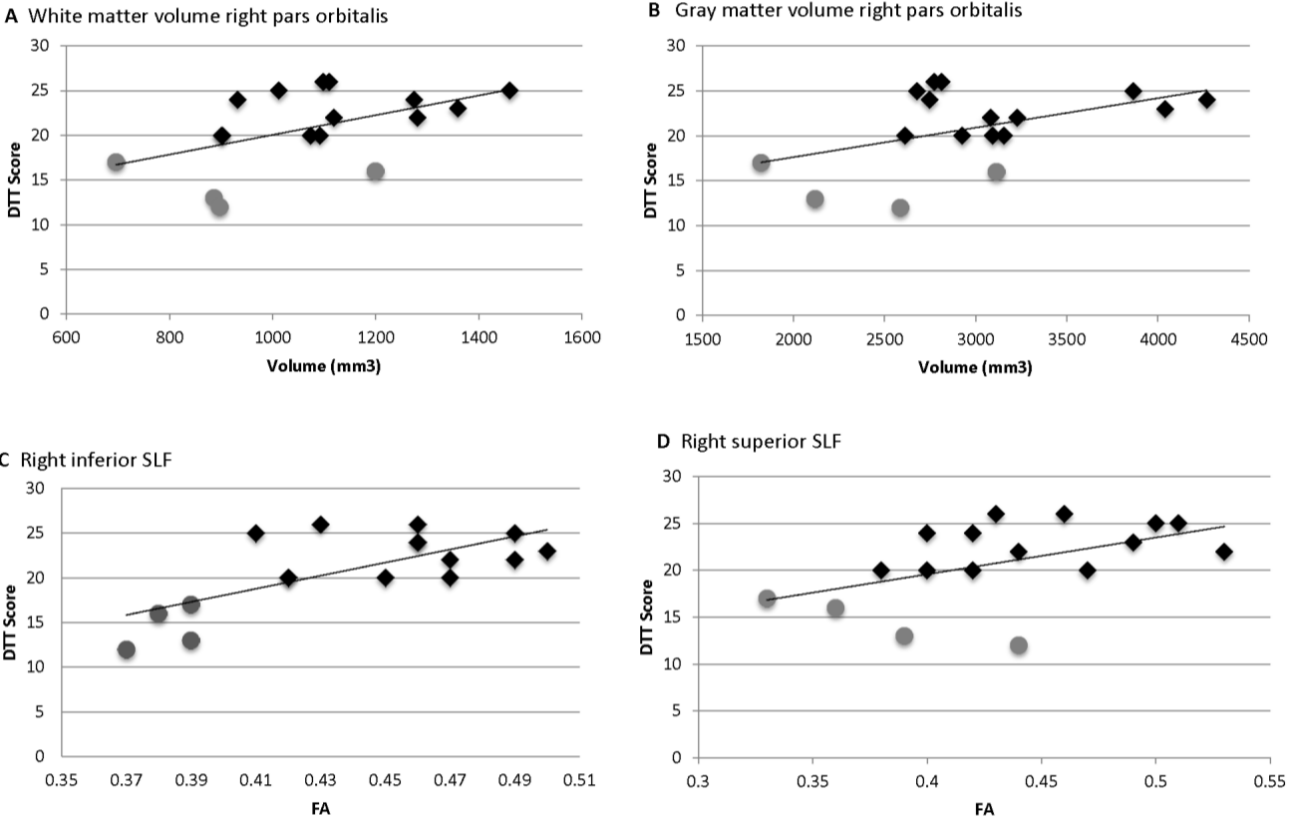
Correlations of Distorted Tunes Test (DTT) scores with brain volume and fractional anisotropy (FA). DTT scores ≤ 18 are considered amusic. ♦ = Non-amusia; 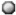 = Amusia. (**A**) DTT scores and white matter volume of right pars orbitalis (*r* = 0.507, *p* = 0.038); (**B**) DTT scores and gray matter volume of right pars orbitalis (*r* = 0.479, *p* = 0.052); (**C**) DTT scores and FA of right inferior SLF (*r* = 0.694, *p* = 0.002); (**D**) DTT scores and FA of right superior SLF (*r* = 0.506, *p* = 0.038).

The zero-order correlations of the volumetric data revealed that Distorted Tunes Test (DTT) score was significantly correlated with white matter volume in the right pars orbitalis (*r* = 0.507, *p* = 0.038; see Figure 3A). This effect remained significant when controlling for sensitivity to specific sounds (*r* = 0.504, *p* = 0.046) or sound characteristics (*r* = 0.504, *p* = 0.047), was marginal after controlling for cumulative years of training (*r* = 0.477, *p* = 0.062), and was not significant when controlling for IQ (*r* = 0.398, *p* = 0.127) or lesson exposure (*r* = 0.184, *p* = 0.495). DTT scores were also marginally associated with gray matter volume in the right pars orbitalis (*r* = 0.479, *p* = 0.052; Figure 3B). While this relationship remained significant after controlling for cumulative years of training (*r* = 0.525, *p* = 0.037), it was marginal after controlling for sensitivity to specific sounds (*r* = 0.473, *p* = 0.064) or sound characteristics (*r* = 0.475, *p* = 0.063), and not significant when controlling for IQ (*r* = 0.343, *p* = 0.194) or lesson exposure (*r* = 0.276, *p* = 0.300). Additionally, though zero-order correlations did not reveal a significant relationship between DTT score and gray matter volume of left pars orbitalis, this relationship was significant when controlling for cumulative years of training (*r* = 0.523, *p* = 0.032) but not when controlling for other potential confounds. Similarly, there was a significant relationship between DTT score and left (*r* = 0.560, *p* = 0.024) and right (*r* = 0.543, *p* = 0.03) superior temporal gyrus gray matter volume only when controlling for sensitivity to specific (non-musical) sounds but not when controlling for other potential confounds. No other significant relationships between DTT and gray or white matter volumetric data were found in the *a priori* regions of interest.

Zero-order correlations between DTT and the fractional anisotropy (FA) data revealed that DTT scores were significantly correlated with FA in the right inferior portion of the superior longitudinal fasciculus (SLF; *r* = 0.694, *p* = 0.002; see Figure 3C) and right superior portion of the SLF (*r* = 0.506, *p* = 0.038; see Figure 3D). The relationship between DTT score and FA in the right inferior SLF remained significant when controlling for IQ (*r* = 0.669, *p* = 0.005), cumulative years of training (*r* = 0.759, *p* = 0.001), sensitivity to specific sounds (*r* = 0.707, *p* = 0.002), sensitivity to sound characteristics (*r* = 0.718, *p* = 0.002), or lesson exposure (*r* = 0.630, *p* = 0.009). The relationship between DTT score and FA in the right superior SLF remained significant when controlling for sensitivity to specific sounds (*r* = 0.592, *p* = 0.016) or sensitivity to sound characteristics (*r* = 0.572, *p* = 0.021) but did not remain significant when controlling for other variables in the model. Additionally, DTT score was significantly correlated with FA in the left superior SLF when controlling for cumulative years of training (*r* = 0.515, *p* = 0.041), sensitivity to specific sounds (*r* = 0.516, *p* = 0.041), or sensitivity to sound characteristics (*r* = 0.511, *p* = 0.043) but not when controlling for other variables.

Preliminary dichotomous analyses comparing the 13 non-amusics to the 4 amusics revealed that the amusics had significantly less gray matter volume in the right (U = 8.0, *p* = 0.023) and left (U = 7.0, *p* = 0.032) pars orbitalis, and there was a trend toward less white matter volume in the right pars orbitalis (U = 9.0, *p* = 0.06). Amusics also had reduced FA in the right inferior (U = 0.0, *p* = 0.001) and superior (U = 8.0, *p* = 0.045) SLF. Following Bonferroni correction, only the group difference in FA in the right inferior SLF remained.

## 4. Discussion and Conclusions

Similar to amusia in TD individuals [7,8,9], we found that amusia in WS, as measured by performance on the DTT, appears to be primarily related to reduced connectivity along the right SLF, a fiber pathway that connects the temporal and frontal lobes [30]. In particular, FA in the right inferior SLF is correlated with DTT score even when controlling for IQ or musical training. In contrast, DTT performance was not associated with gray or white matter volume of the transverse temporal gyri, suggesting that performance on the amusia measure is not simply due to difficulties with pitch discrimination.

Previous research in WS has focused on associations between auditory cortex volumes and specific auditory skills. Martens *et al.* [24] reported that gray matter volume of the left planum temporale was greater in 6 individuals with WS who performed well (*i.e.*, at the level of TD controls) on a series of melodic and rhythmic musical production tests *versus* 14 participants with WS who demonstrated impairments on these tasks. In particular, left planum temporale volume was associated with participants’ abilities to sing the final note of a melodic phrase, a task which involves both perception and production abilities. However, lack of information about the participants’ musical training and the method for measuring performance on the musical tasks limit the generalizability of these findings. Wengenroth *et al.* [25] reported that increased leftward asymmetry of Heschl’s gyrus in WS was associated with a holistic auditory processing style (*i.e.*, perceiving complex sounds based on the fundamental frequency rather than harmonic overtones). Again, the musical training of the WS participants was not well characterized, as the participants were described as having “only minor musical training” and being matched to the TD group. Findings from the present study suggest that previous volumetric studies may have been limited by a focus on regions associated with the primary auditory cortex rather than the entire network involved in auditory processing. Indeed, functional neuroimaging studies have reported more diffuse activation to music throughout the neocortex in WS *versus* TD controls, including the frontal lobes [68,69].

Beyond adding to our knowledge about the variability of musical skills in individuals with WS, the present study adds to a growing body of literature on the neural basis of amusia in general. Our finding of reduced FA in the right inferior SLF in individuals with amusia in WS is highly consistent with converging evidence from structural and functional MRI, DTI, and ERP studies of amusia in TD. Specifically, this work suggests that amusia is due to disconnectivity in the right fronto-temporal pathway connecting the right auditory cortex and inferior frontal lobe rather than isolated dysfunction in the primary auditory cortex [7,8,9,10,15,16,35]. These previous studies also documented decreased white matter in the pars orbitalis [7] and decreased volume and FA in the right arcuate fasciculus [8] in amusia, with volume of these structures correlating with performance on melodic pitch, pitch discrimination, and pitch perception-production tasks. This suggests that fronto-temporal disconnectivity underlies difficulties with awareness, encoding, and maintenance of pitch information in amusia, which is needed for melodic perception and production activities. The current study similarly found that decreased white matter in the right pars orbitalis was associated with performance on the DTT in WS, though this relationship did not remain significant when controlling for IQ or musical training and thus may be related to other cognitive and perceptual factors.

While the relationship between amusia and right fronto-temporal disconnectivity was present for both the inferior and superior components of the SLF in the present study, it was particularly strong in the inferior SLF and remained significant after controlling for cognitive abilities or musical training. A benefit of using ROQS comes from the fact that ROI selection and FA analysis are performed on an individual basis. This avoids potential confounds that can be introduced by non-linear spatial normalization techniques [67]. The semi-automated tract selection employed by ROQS does not define ROIs strictly on an anatomical basis, making it more difficult to directly compare FA from inferior *versus* superior SLF to values in the published literature. Nevertheless, we can make conjectures about how the inferior and superior portions of the SLF in the current paper compare with the inferior and superior components of the arcuate fasciculus examined in a previous study of connectivity in TD amusics.

Loui *et al.* [8] defined the inferior arcuate fasciculus as the white matter connecting the posterior IFG to the posterior middle temporal gyrus, while the superior arcuate fasciculus was defined as the white matter connecting the posterior IFG to the posterior superior temporal gyrus. Interestingly, volume in the right superior arcuate fasciculus correlated with pitch perception thresholds, while volume in the right inferior arcuate fasciculus correlated with the degree of mismatch in auditory perception and vocal production. In the DTT task used to index amusia in the current study, participants had to recognize out-of-tune notes in familiar melodies. Thus, while the DTT is predominantly a perception task, it is possible that participants compared the melodies with internally generated models of the actual (in tune) melodies, such that the demands of the DTT extend beyond basic pitch discrimination. Previous work in WS has demonstrated that variability in DTT performance accounts for nearly one-quarter of the variability observed in singing skill, suggesting that perception abilities on the DTT are closely linked with musical production abilities [17]. In broad terms, therefore, our disconnectivity results in WS appear consistent with previous findings in TD individuals with amusia.

It is noteworthy that while the correlation between amusia and right SLF withstood corrections for variance due to IQ and musical training, the relationship between amusia and white matter volume of the right pars orbitalis did not. Amusia may reflect an impairment in auditory-motor feedback or integration, such that amusics are unable to use auditory feedback to guide musical motor behaviors (such as singing [8,36]). Previous research has demonstrated that perception abilities (on the DTT) significantly predicted singing abilities in WS (much more so than musical training [17]). Furthermore, explicit awareness of using auditory/auditory-motor learning strategies predicted skill acquisition on a new musical instrument in WS beyond prior musical or motor skills, highlighting the link between auditory and motor processes in developing musical abilities in a developmental disorder [39]. Intact fronto-temporal connectivity may be critical for music learners to consciously translate musical training experiences into musical skill through use of auditory and auditory-motor feedback.

Though the current results in WS were predominantly right lateralized, reduced gray matter volume in the left pars orbitalis and reduced FA in the left superior SLF were found when controlling for cumulative years of training (but not when controlling for other cognitive, perceptual, or musical training factors). Similarly, though the aggregated findings from studies in TD individuals suggest that the neural correlates of amusia are predominantly right lateralized, bilateral differences in cortical thickness have been reported in the IFG and auditory cortex of amusics [35], as well as decreases in left hemispheric gray matter volume in these regions [36]. Additionally, abnormally increased functional connectivity between right and left auditory cortices in amusics has been reported [9]. It may be that individuals with amusia show developmental differences bilaterally but that right hemispheric differences are more severe and thus a more reliable indicator of amusia. It is also possible that right fronto-temporal differences are related to a cascade of connectivity-based structural changes across both hemispheres. Supporting this idea, Hyde *et al.* [35] suggest that the thicker cortex found in amusia is related to abnormal neuronal migration. Increased cortical thickness has been reported in the right [28] and left [70] auditory cortices in WS, while histological analyses of three brains from individuals with WS suggest atypical asymmetries in cell packing density and cell size in specific layers of the auditory cortex [26].

Additionally, our results revealed an association between poorer pitch performance on the DTT and reduced gray matter volume in the superior temporal gyri bilaterally but only when controlling for variance due to general auditory sensitivities (and not when controlling for other cognitive or musical training factors). Recent studies of TD individuals with amusia also find reduced gray matter volume [10] and increased cortical thickness [35] in this region. It is interesting that the association between DTT performance and superior temporal gyri volume only emerged in WS when controlling for general auditory sensitivities given the heightened auditory sensitivities in WS [20,41] and the lack of association between general auditory sensitivities and amusia in the present study and in a larger sample of individuals with WS [17]. Functionally, previous studies in TD individuals with amusia have observed that the auditory cortex is unimpaired for simple acoustic tasks but not for more complex auditory processing [10,15,16]. These findings are in line with the idea that amusia is not simply due to difficulties with pitch discrimination but instead related to impairments in encoding and maintaining melodic contour information in memory [10].

Additionally, these findings that superior temporal gyri volume only correlated with DTT performance when controlling for auditory sensitivities may further our understanding of the heightened emotional responsiveness to music in WS [21,22]. Previous work has indicated small to moderate associations between performance on the DTT and musical interest and emotional responsiveness in a large sample of individuals with WS [17], consistent with the reduced musical engagement and emotional reactions in TD individuals with amusia (e.g., [71,72]). However, follow-up analyses revealed that the WS participants’ general auditory sensitivities, and not their pitch perception abilities as measured by the DTT, predicted their emotional responsiveness to music, suggesting that the physical or acoustic qualities of music (*i.e.*, timbre, dynamics, articulation, *etc.*) may be more important than tonal expectancies for the emotional connection to music in WS [17]. Indeed, an fMRI study indicated greater activation in bilateral superior temporal gyri in response to emotional music in participants with WS *versus* TD individuals [69], which may have been related to processing these physical attributes of music.

The consistency of the neural basis for amusia in WS with previous results from TD individuals suggests that WS may be a model system for further exploration of the roles of genes and environment in the development of musical skill. Studies of congenital amusia in TD individuals have hypothesized that amusia is due to anomalous neural development rather than a lack of exposure to musical experiences and/or training. Individuals with WS typically have as much, if not more, musical exposure than TD individuals with regard to their musical listening and playing habits [21]. In a previous study, we demonstrated that while (a lack of) formal musical training was related to amusia in WS, time spent listening to or playing music was not related to performance on an amusia measure [17]. In the current study, the relationship between fronto-temporal connectivity and performance on the DTT in WS remained significant when controlling for musical training, adding to the evidence that amusia is not simply due to a lack of musical experiences.

Studying amusia in WS may also yield an increased understanding of the specificity of the neural correlates of amusia. For example, previous studies have suggested that amusia is associated with deficits in visual-spatial perception [73], although a subsequent study did not find evidence for a relationship between the pitch and spatial domains [74]. A hallmark of WS is a deficit in visual-spatial abilities, particularly visual-spatial construction [75]. Additionally, an inverse relationship has been reported between FA in the right SLF and visual-spatial construction in WS [33]. Future studies could examine visual-spatial perception and construction in both WS and TD amusics and non-amusics to elucidate whether or not these pitch and spatial domains are necessarily functionally linked.

Researchers have also suggested that there may be a shared neural basis for amusia and language difficulties such as dyslexia [76]. Loui and colleagues [76] reported an association between pitch perception-production discrepancies and phonemic awareness in children. In a TD adult sample, poor pitch perception on the DTT was associated with weaker phonological and phonemic awareness [77]. Impaired performance on certain phonologic tasks, as well as weaker relationships between these pre-reading and reading abilities, have been reported in WS compared to TD individuals (e.g., [78,79,80]). Therefore, examining these constructs in WS may help elucidate these functional relationships and their neural underpinnings.

The current study uniquely extends the neural basis of amusia to a known genetic syndrome. Additional strengths of the study include the use of both volumetric and connectivity neuroimaging measures; the use of individual-specific (versus group-averaged) data via the use of ROQS; the well-characterized musical background of the sample; and the exploration of relationships while controlling for cognitive, auditory sensitivity, and music training factors. Furthermore, we quantified musical training in ways that are both comparable to the TD literature yet meaningfully reflective of the training experiences of individuals with developmental disabilities. Future studies can extend this work by using additional measures of amusia beyond the DTT, including tests of pitch discrimination thresholds and musical production abilities, as well as basic hearing assessments such as pure tone thresholds. Studies could also examine how amusia in WS relates to their short-term and working memory capacities across different auditory tasks given the pitch specific memory deficits in TD amusics [81]. Future studies also could strive to define anatomically distinct sub-tracks of white matter ROIs, unlike those obtained with ROQS, which would permit more direct comparisons to the anatomical literature.

The challenges of conducting neuroimaging studies in a rare syndrome such as WS have been noted previously [25,69]. The small sample size, particularly of individuals with WS with amusia, in the current study may have precluded our ability to detect significance for smaller (but potentially relevant) relationships. Similarly, a larger sample size would allow for an exploratory whole brain analysis to investigate other potential ROIs. Consistent with Wengenroth *et al.* [25], however, our use of a hypothesis-driven approach, individual-specific data, and the robustness of findings in regards to the right inferior SLF, somewhat tempers these concerns. Nevertheless, future studies can aim to enroll larger numbers of individuals with WS with amusia, which would enable a more thorough investigation of the phenomenon of amusia and its neural correlates in WS. Finally, our sample was cross-sectional, whereas longitudinal studies would permit the investigation of how amusia unfolds over the course of development in WS. Overall, however, our current findings in WS are remarkably consistent with previous studies of amusia in TD individuals, which document fronto-temporal disconnectivity as the neural marker for amusia. The current results expand upon previous behavioral findings of amusia in WS and highlight the value of examining individual differences in amusia in novel populations as a window into gene-brain-behavior relationships.

## Supplementary Files

Supplementary File 1
